# The effect of corn trypsin inhibitor, anti-tissue factor pathway inhibitor antibodies and phospholipids on microvesicle-associated thrombin generation in patients with pancreatic cancer and healthy controls

**DOI:** 10.1371/journal.pone.0184579

**Published:** 2017-09-14

**Authors:** Marit Hellum, Isabel Franco-Lie, Reidun Øvstebø, Truls Hauge, Carola E. Henriksson

**Affiliations:** 1 Institute of Clinical Medicine, University of Oslo, Oslo, Norway; 2 Department of Medical Biochemistry, Oslo University Hospital, Oslo, Norway; 3 Department of Gastroenterology, Oslo University Hospital, Oslo, Norway; Institut d'Investigacions Biomediques de Barcelona, SPAIN

## Abstract

Circulating microvesicles (MVs) are suggested to be important contributors to cancer-associated thrombosis due to the presence of surface-bound procoagulant molecules like tissue factor (TF) and phosphatidylserine (PS). Pancreatic cancer is considered to be one of the most prothrombotic malignancies. The aim of this study was to describe the impact of analytical variables on MV-associated thrombin generation in patients with pancreatic cancer and in healthy controls. MVs were isolated from citrated plasma and added to pooled normal plasma (PNP). Thrombin generation was measured by the calibrated automated thrombogram. The impact of corn trypsin inhibitor (CTI), anti-tissue factor pathway inhibitor (TFPI) antibodies and phospholipids was described. Antibodies against TF were used to assess TF-dependency, and MV-bound PS activity was measured with the Zymuphen MP-activity kit. MVs from the pancreatic cancer patients displayed higher thrombin generation and higher PS-activity than MVs from the healthy control group, while TF-dependency was observed in only 1 out of 13 patient samples. Adequate thrombin generation-curves were only achieved when CTI was omitted and anti-TFPI antibodies were added to PNP prepared in low contact-activating tubes. Addition of phospholipids reduced the significant differences between the two groups, and should be omitted. This modified thrombin generation assay could be useful for measurement of procoagulant circulating MVs, allowing the contribution from MVs affecting both the intrinsic and the extrinsic pathway to be measured.

## Introduction

Cancer patients are at increased risk of developing venous thromboembolism (VTE), and growing evidence suggests that circulating microvesicles (MVs) are important contributors to the prothrombotic state [1, 2]. MVs are small membrane vesicles (< 1 μm) that are shed from a variety of cells as a response to either activation or apoptosis [3], and they may carry the procoagulant negatively charged phospholipid phosphatidylserine (PS) on their surfaces. MV-associated PS provides a catalytic surface for assembly of coagulation factors in general, and it also acts in synergy with tissue factor (TF) that may be present on the MVs [4, 5]. TF is the main initiator of coagulation [6], and also a promotor of tumor cell growth, angiogenesis and metastasis [7]. Tumor cells of a variety of cancers are reported to express TF [8–10], and circulating, tumor-derived MVs carrying TF have been linked to cancer-associated thrombosis (reviewed in [11]). Pancreatic cancer is considered to be a highly prothrombotic malignancy [12], and elevated levels of MV-associated TF-activity in these patients have been associated with an increased risk of mortality [13, 14], a more aggressive, invasive phenotype [15] and VTE [14, 16–18].

At present, the reports of PS/TF-positive MVs as biomarkers for VTE in cancer are inconsistent, to which methodological limitations and lack of standardization probably contribute considerably [2, 11, 19–21]. The methods used to measure TF-activity of circulating MVs in plasma are chromogenic factor Xa (FXa)-generating assays, or plasma-based thrombin- or fibrin generation assays in combination with inhibiting anti-TF antibodies (abs) (reviewed in [22]). The calibrated automated thrombogram assay (CAT) was originally developed by Hemker *et al*. for measuring thrombin generation in platelet-rich or platelet-poor plasma in a variety of clinical settings [23]. In a recent prospective study on patients with different cancer types, the thrombin generation peak in plasma was used to identify patients with an increased risk of VTE [24]. A modified thrombin generation assay, where MVs in plasma are pelleted, plasma removed and MVs resuspended in pooled normal plasma (PNP), has also been used in several studies [25–27]. In such a modified assay, the measurement of thrombin generation will not be affected by the presence of anticoagulants or a consumptive coagulopathy. Both endogenous phospholipids, by means of MV-associated PS, and externally added phospholipids (i.e. PS-containing MP-reagent) may contribute to the total thrombin generation in CAT. Addition of a phospholipid-containing MP reagent is recommended by the suppliers to make MV-associated thrombin generation more sensitive to low amounts of TF in the sample. Additionally, at low levels of TF, the use of corn trypsin inhibitor (CTI) is recommended to eliminate the contribution from unspecific contact activation of factor XII (FXII) [28].

Other ways to modulate the thrombin generation is to target plasma inhibitors [29, 30]. Tissue factor pathway inhibitor (TFPI) is the primary, physiologic inhibitor of the initiation of blood coagulation. The TFPIα isoform binds to FXa, and this TFPI/FXa complex binds to and inhibits the TF-factor VIIa (TF-FVIIa) complex [31]. The presence of TFPI in plasma might inhibit the measurement of low levels of TF [22], and the addition of anti-TFPI antibodies (abs) to plasma has been used to increase the sensitivity of TF in MV-associated thrombin generation [29, 32]. Due to their diverse composition, MVs may impact the coagulation differently, and optimal analytical conditions for measuring MV-associated thrombin generation may vary between disorders.

The aim of this study was to describe the effect of CTI, anti-TFPI abs and phospholipids (MP-reagent) on the MV-associated thrombin generation in samples from patients with pancreatic cancer and healthy controls, and find the best conditions to compare these two groups.

## Material and methods

### Ethics approval

The project was approved by the Regional Committee for Medical and Health Research Ethics, South-Eastern Norway (2015/1124/REK sør-øst C) and by the Data Protection Supervisor at Oslo University Hospital (2015/9517). The patients were originally included in the Thematic Pancreatic Tumour Project (TPTP), which was approved by the Regional Committee for Medical and Health Research Ethics, South-Eastern Norway (265–08412 C), the Norwegian Directorate of Health (08/7927) and The Norwegian Data Protection Authority (08/01409-2).

### Patients and blood collection

#### Pancreatic cancer patients and healthy controls

The patients were originally included prospectively in the Thematic Pancreatic Tumour Project (TPTP) between January 1^st^ 2012 and September 30^th^ 2013. Citrated plasma samples were available for 14 of these patients. The inclusion criteria for patients in the present study was newly diagnosed and pathologically confirmed (cytology and/or histology) pancreatic cancer and absence of another previous (the last five years) cancer diagnosis. Among the 14 patients one was excluded due to concomitant diagnosis of another cancer. The group included eight men and five women and the mean age at diagnosis was 64.1 years (SD 7.3). Among the 13 patients, four used acetylsalicylic acid (as prophylaxis of cardiovascular disease) and one used warfarin due to several previous venous thromboembolisms. None of the patients had started with chemotherapy or used hormone therapy. The controls (n = 13) were recruited, in retrospect, among healthy candidates that matched the patients by age (+/- five years) and gender. None of the controls used anticoagulants. All study subjects (patients and controls) gave their written informed consent, and blood was drawn without requirements of fasting. Patient plasmas were collected in 2012–2013 and plasmas from healthy controls were collected in 2015. Citrate-anticoagulated blood (0.109 M, Vacuette, GreinerBioOne Gmbh, Kremsmünster, Austria) was collected by venipuncture (21G needle) and the first tube was discarded. Plasma was prepared at 2,500 g, 15 min, room temperature (RT) within one hour after blood collection, and then aliquoted and stored frozen at -80°.

### Reagents

Tris buffered saline (TBS) and bovine serum albumin (BSA) were purchased from Sigma Aldrich (St. Louis, MO, USA). Thrombin Calibrator, MP-reagent (4 μM phospholipids) and FluCa-kit were from Thrombinoscope BV (Maastricht, the Netherlands). The anti-human TFPI abs (CLB/TFPI C-terminus) was from Sanquin Reagents (Amsterdam, the Netherlands). CTI was purchased from Enzyme Research Laboratories (IN, USA). The hybridoma cells TF8-5G9 were a kind gift from professor James H.Morrissey (University of Illinois, College of Medicine, Urbana, USA), and The Core Facility for monoclonal antibody production and assay design (Department of Medical Biochemistry, Oslo University Hospital, Oslo, Norway) produced the TF8-5G9 anti-TF abs.

### Isolation of MVs

Citrated plasma (>500 μL) from pancreatic cancer patients and healthy controls was thawed (once) for 15 min at 37°C, and then 500 μL plasma was transferred to a new tube and spun to pellet the MVs (17,000 g, 30 min, RT). Subsequently, 450 μL plasma was removed, and the remaining MV-enriched pellet was washed with 450 μL tris-buffered saline with 0.5% v/v bovine serum albumin (TBSA), briefly vortexed and centrifuged again (17,000 g, 30 min, RT). The supernatant was removed, and the MV pellet was dissolved in 50 μL TBSA and vortexed for 5 sec.

### Pooled normal plasma (PNP)

Blood was collected in Monovette tubes (0.106 M citrate, 5 mL, Sarstedt, Nümbrecht, Germany) with and without manually prefilled CTI (18.3 μg/mL final concentration). The venipuncture was performed with a Safety-Multifly®-Needle 21G (Sarstedt, Nümbrecht, Germany), and the first tube was discarded. After written informed consent, eight healthy volunteers donated blood to four tubes with CTI and four tubes without CTI. The tubes were centrifuged (2,000 g, 15 min, RT) and pooled into two separate plasmas (+/- CTI) which were subjected to another centrifugation at 2,000 g, 15 min, RT. The PNP (+/- CTI) was aliquoted and stored at -80°C. At the day of use, the PNP (+/- CTI) was thawed at RT, and incubated (15 min at 37°) with anti-human TFPI abs (100 μg/mL final conc.) or an equal volume of TBSA before addition to the wells. The concentration of anti-TFPI abs was chosen based on initial *in vitro* thrombin generation experiments, where 100 μg/mL of anti-TFPI abs gave the highest sensitivity for detection of MVs obtained from whole blood stimulated with *Neisseria meningitidis* bacteria.

### MV-associated thrombin generation (calibrated automated thrombogram, CAT)

The ability of plasma-derived MVs to generate thrombin was measured with a modified version of the CAT originally described by Hemker *et al* [23]. Seventy μL PNP (+/- CTI) with or without anti-TFPI abs was added to wells (Thermo Immulon 2HB plate, Thermo Scientific) containing either: (20 μL MP-reagent + 10 μL MVs), (20 μL TBSA + 10 μL MVs) or (20 μL thrombin calibrator + 10 μL TBSA). To test for TF-dependency, in parallel wells, MVs were incubated with 2 μL of a mouse monoclonal anti-TF antibody TF8-5G9 (0.40 mg/mL) for 15 min (RT) before addition of the PNP. All samples were run in duplicate. Since we use the same PNP, all samples were related to the same calibrator (+/- anti-TFPI abs). The plate was incubated for 10 min at 37°C before the reaction was initiated by automated addition of Fluobuffer containing calcium and a fluorogenic substrate. During 90 min, fluorescence was read by a Fluoroscan Ascent machine (Thermo Scientific, MA, USA). The thrombin generation parameters lag time (LT), peak, endogenous thrombin potential (ETP) and time to peak (ttPeak) were calculated by the Thrombinoscope software (Thrombinoscope BV, Maastricht, the Netherlands). Velocity index was calculated as peak (nM thrombin)/(ttpeak (min)-lag time (min)), and represented the rate of thrombin generation during the propagation phase [33]. Of note, if the curve did not reach the baseline within 90 min, the ETP was not calculated, and the peak will be overestimated due to the lack of α-2-macroglobulin correction. If a curve did not appear during this period, we appointed the LT to 90 min for calculation purposes.

### Zymuphen MP-activity assay

Citrated plasma from pancreatic cancer patients and healthy controls was thawed (for a second time) at 37°C for 15 min. The activity of phosphatidylserine (PS) in the plasma (1:50 dilution) was measured with the Zymuphen MP-activity assay (Hyphen BioMed, Neuville-sur-Oise, France). Briefly, PS-positive MVs are captured on an ELISA plate coated with annexinV-streptavidin. In the presence of FVa/FXa and calcium, captured MVs with negatively charged phospholipids cleave prothrombin to thrombin, and the phospholipid concentration is the rate-limiting step. Finally, a chromogenic substrate for thrombin is added to assess the levels of thrombin. The results are expressed as PS-equivalents (nM).

### Statistics

All results are presented as mean (SD), and comparison of means were performed with repeated measures (RM) two-way ANOVA with Sidak’s multiple comparison test, or an unpaired t-test with Welch correction (PS-equivalents). Associations between thrombin generation parameters and PS equivalents were evaluated with Pearson’s test. All tests were performed with GraphPad Prism version 6.01. Repeatability is reported as coefficient of variation (CV) from duplicate measurements, made across a number of different experiments and subjects (S1 Table).

## Results

### The effect of MP-reagent and anti-TFPI abs on MV-associated thrombin generation in PNP with CTI

**Fig 1:**
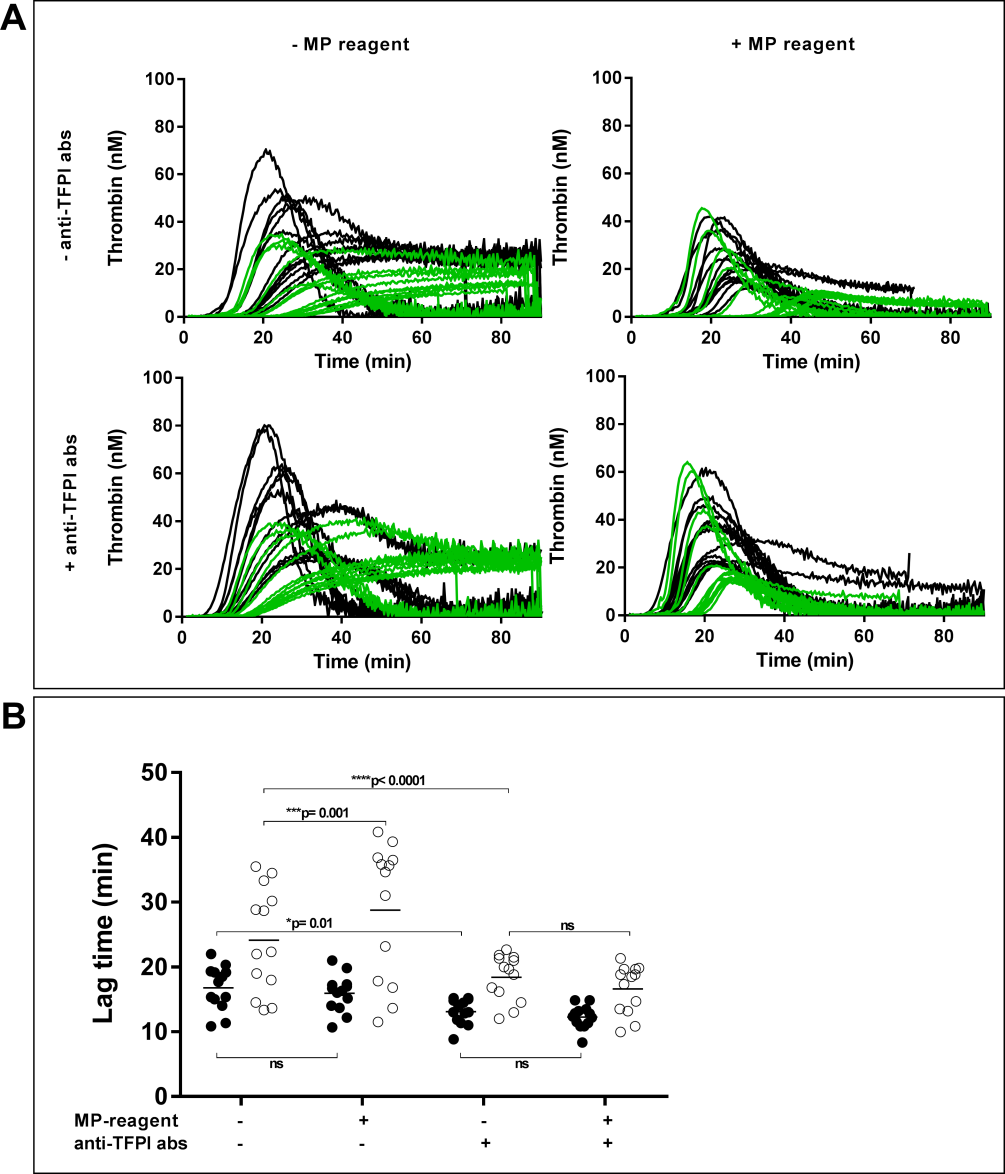
Microvesicle (MV)-associated thrombin generation in pooled normal plasma (PNP) with corn trypsin inhibitor (CTI). MVs were isolated from plasma, washed (17,000 g, 30 min, RT) and added to CTI-containing PNP. Thrombin generation was measured -/+ MP-reagent (phospholipids) and -/+ anti-TFPI antibodies (abs). A) Thrombin generation curves of MVs from pancreatic cancer patients (black) and healthy controls (green). B) Lag time (LT) of MVs from pancreatic cancer patients (filled circles) and healthy controls (open circles). Lines indicate mean LT, and * indicates significant differences (RM two-way ANOVA with Sidak’s multiple comparison test, adjusted p-values are shown).

The PNP (with 18.3 μg/mL CTI) itself did not generate any thrombin during 90 min of recording. MVs isolated from plasma from patients with pancreatic cancer and healthy controls generated small amounts of thrombin when added to this PNP (Fig 1A). Most of the thrombin generation curves did not reach the baseline, which excluded the correct estimation of other parameters except for the LT. Repeatability, reported as coefficient of variation (CV) from duplicate measurements, during native thrombin generation (i.e. measurements without the addition of an MP-reagent and anti-TFPI abs) was 8% for LT < 20 min (n = 33), and 17% for LT > 20 min (n = 19) (S1 Table).

Addition of anti-TFPI abs shortened the LTs significantly in both the pancreatic cancer and the healthy control group, and it also reduced the variability between individuals in both groups, most evidently within the healthy group (Fig 1B). The CV of duplicate measurements in the presence of anti-TFPI abs was 6% at LT < 20 min (n = 40), and 12% at LT > 20 min (n = 12) (S1 Table). Both in the absence and presence of anti-TFPI abs in the PNP, addition of an MP-reagent (4 μM phospholipids) did not affect the mean LT in either group, except for the statistically prolonged LT in the healthy control group in the absence of anti-TFPI abs (Fig 1B). Generally, adding external phospholipids reduced the differences of the overall thrombin generation curves between the pancreatic cancer and the healthy control group (Fig 1A, compare the two charts on the right with the two charts on the left).

**Fig 2:**
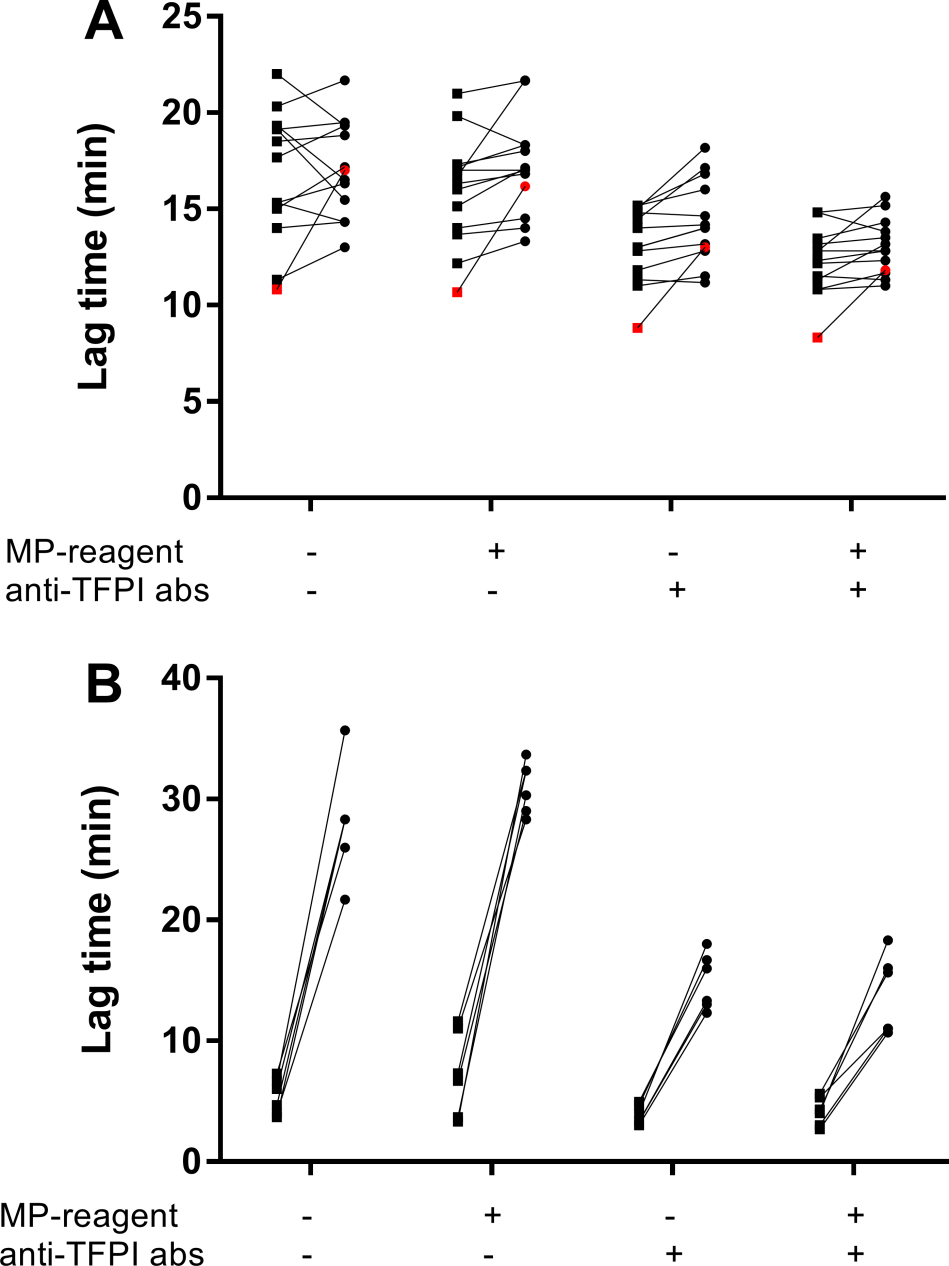
The effect of inhibiting antibodies against tissue factor (TF) on microvesicle (MV)-associated thrombin generation in pooled normal plasma (PNP) with corn trypsin inhibitor (CTI). The thrombin generation (lag time) of MVs isolated from pancreatic cancer patients plasma (A) or of MVs isolated from plasma obtained from citrated whole blood that had been incubated with *Neisseria meningitidis* (10^8^/mL) (B). MVs were analyzed with (circles) or without (squares) preincubation with anti-TF abs, in the presence or absence of phospholipids (MP-reagent) and antibodies against TFPI (anti-TFPI abs). The patient sample with the shortest lag time and the most evident TF-dependent thrombin generation across the four conditions is marked in red.

To test for TF-dependency, MVs were incubated with anti-TF abs prior to measurements of thrombin generation. As a group, MVs from pancreatic cancer patients did not show an evident TF-dependent thrombin generation (Fig 2A). Notably, the sample with the shortest LT showed a prolonged LT across all assay conditions when preincubated with anti TF-abs, indicating TF-dependency (Fig 2A, marked in red). MVs isolated from plasma obtained from citrated whole blood that had been incubated with *Neisseria meningitidis* (10^8^/mL) for 4 hours served as positive controls (n = 6), and these samples were analyzed in the same run as the patient samples. Consistent across the four assay conditions, the positive controls showed an evident prolongation of the LT after preincubation with anti-TF abs (Fig 2B).

### The effect of MP-reagent and anti-TFPI abs on MV-associated thrombin generation in PNP without CTI

**Fig 3:**
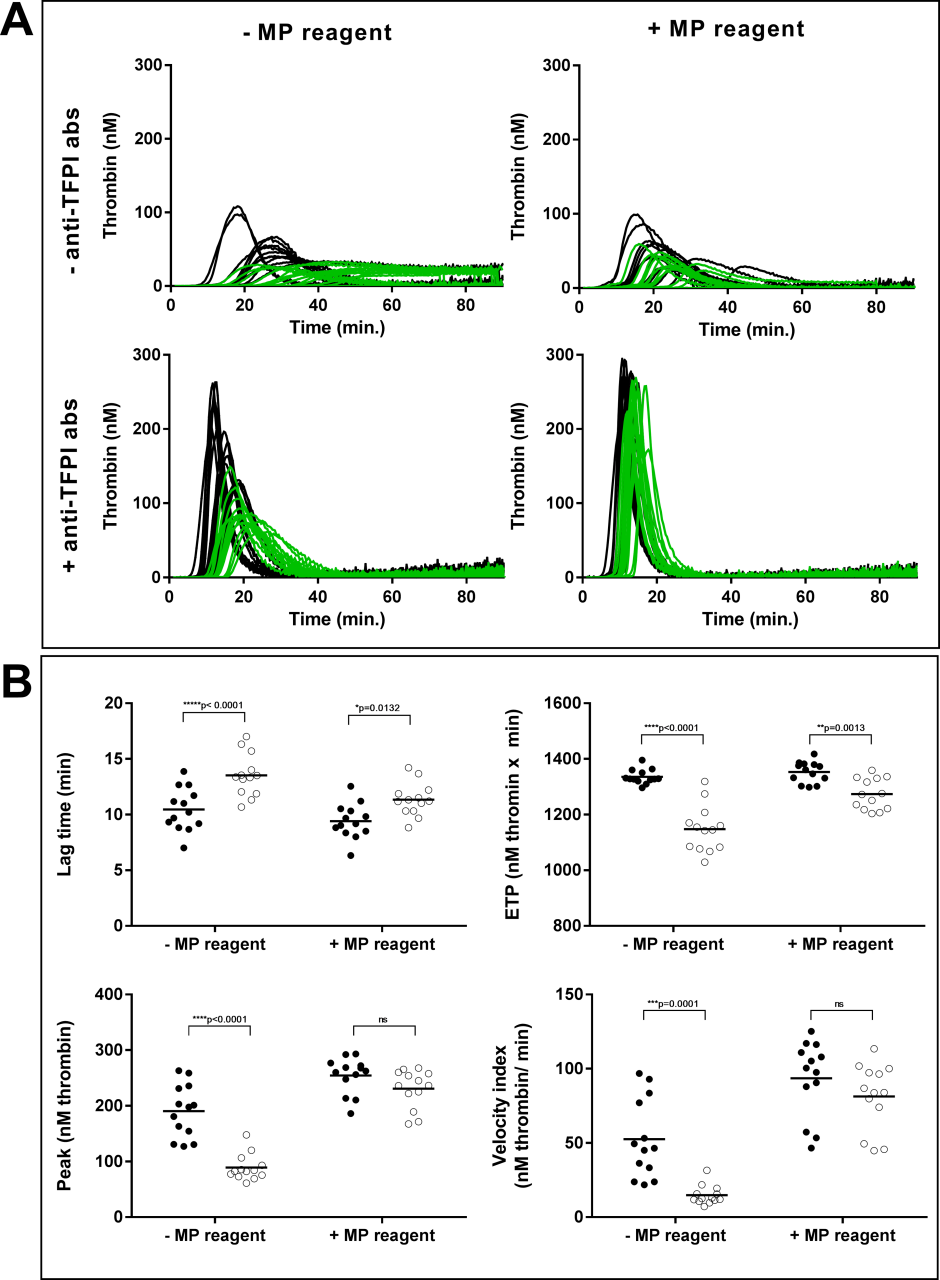
MV-associated thrombin generation in pooled normal plasma (PNP) without corn trypsin inhibitor (CTI). MVs were isolated from plasma, washed (17,000 g, 30 min, RT) and added to PNP without CTI. Thrombin generation was measured -/+ MP-reagent (phospholipids) and -/+ anti-TFPI antibodies (abs). A) Thrombin generation curves of MVs from pancreatic cancer patients (black) and healthy controls (green). B) LT, ETP, peak and velocity index of MVs from pancreatic cancer patients (filled circles) and healthy controls (open circles) in PNP with anti-TFPI abs added. Lines indicate mean values, and * indicates significant differences between the pancreatic cancer group and the healthy control group (RM two-way ANOVA with Sidak’s multiple comparison test, adjusted p-values are shown).

The PNP (without CTI) itself did not generate any thrombin during 90 min of recording. In this PNP, native thrombin generation of MVs from the control group was comparable to what we observed in PNP with CTI, with most of the curves not reaching the baseline (compare the green curves, upper left in Figs 3A and 1A, note the different scales on the Y-axes). On the other hand, the absence of CTI increased the thrombin generation in several of the samples from the pancreatic cancer group (compare the black curves, upper left in Figs 3A and 1A). Visually, the overall shape of the native thrombin generation curves discriminated well between MV-associated thrombin generation of pancreatic cancer patients and healthy controls (Fig 3A, upper left, green and black lines). In PNP without CTI, the CV of duplicate measurements was 15% for samples with LTs < 20 min (n = 14), and 39% for samples with LTs > 20 min (n = 11) (S1 Table).

Addition of anti-TFPI abs in the PNP resulted in increased thrombin generation and curves that reached baselines for all samples (Fig 3A, lower left). This allowed us to evaluate the entire thrombograms, and we found significant differences between the pancreatic cancer group and the healthy control group for all parameters; LT: 10 (2) vs 14 (2) min (p<0.0001), ETP: 1336 (26) *vs* 1147 (83) nM thrombin x min (p<0.0001), peak: 191 (48) *vs* 89 (23) nM thrombin (p<0.0001) and velocity index: 53 (27) *vs* 15 (6) nM thrombin min (p = 0.0001) (Fig 3A (lower left) and 3B (without (-) MP reagent)). For all thrombin generation parameters, adding an MP-reagent in the presence of anti-TFPI abs reduced or eliminated the significant differences between the two groups (Fig 3A (lower right) and 3B (with (+) MP reagent)).

Thus, the addition of anti-TFPI abs to PNP without CTI, and without an MP-reagent, seemed to be the best condition to describe MV-associated thrombin generation in patients with pancreatic cancer and healthy individuals. During these conditions the assays duplicate CVs were 10% (LT), 1.5% (ETP), 6% (peak) and 15% (velocity index) (n = 26 for all parameters, S1 Table).

We wanted to verify that PNP without CTI (-PS, +anti-TFPI abs) would permit detection of TF-dependent thrombin generation. However, in initial experiments, using PNP with CTI, we only detected a clear TF-dependency in 1/13 samples from the pancreatic cancer patients (Fig 2A). Also, we had limited amounts of plasma from these 13 patients. We therefore used MVs isolated from patients with meningococcal sepsis, which we previously have shown to have a highly TF-dependent thrombin generation [34]. Within the sepsis group, the LT was significantly prolonged from 11 (3) min to 17 (3) min when the MVs were incubated with anti-TF abs before the measurements of thrombin generation (n = 7, p = 0.0032, paired t-test, S1 Fig), indicating that TF-dependency could be detected.

### Zymuphen MP-activity assay

**Fig 4:**
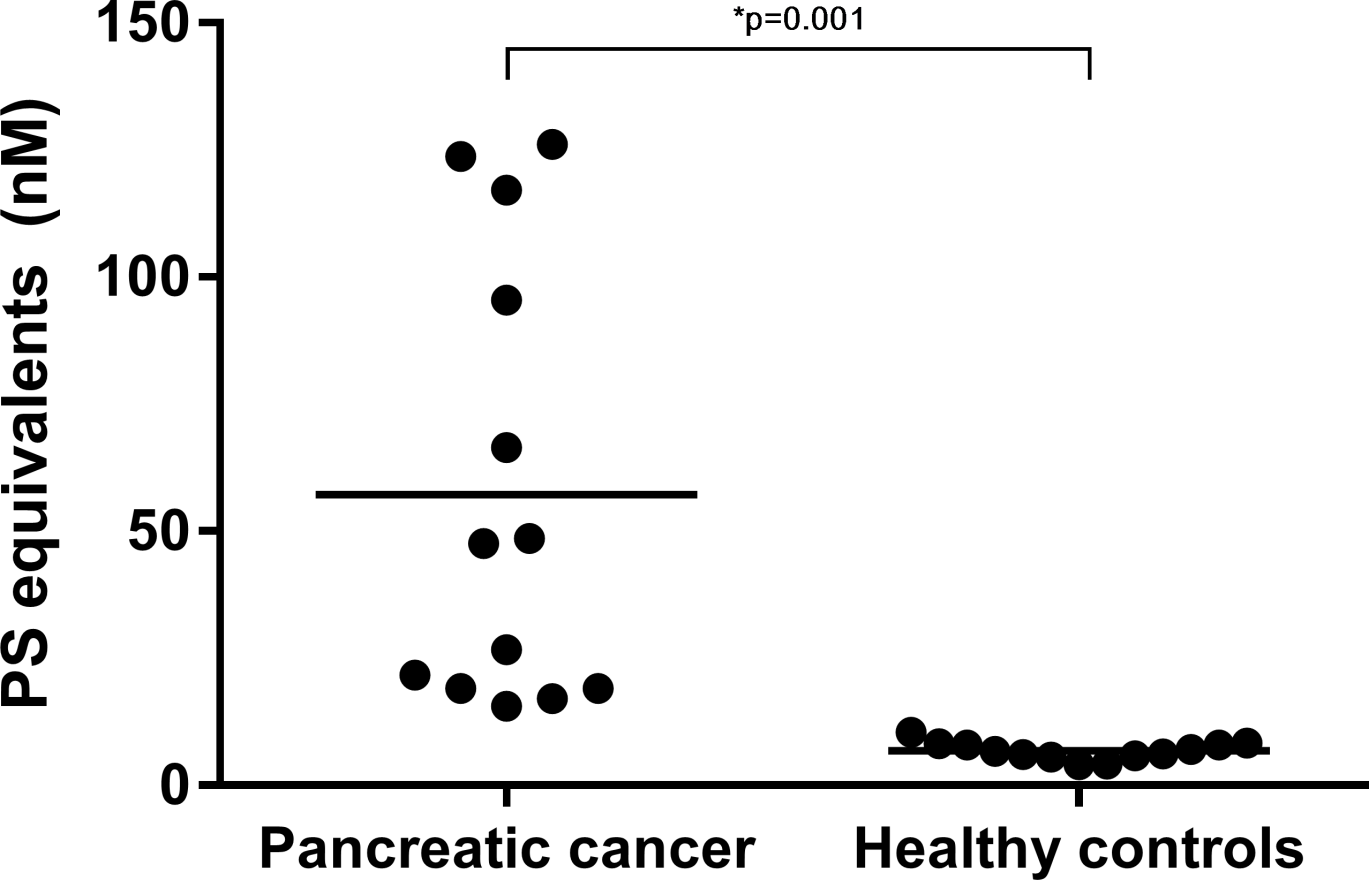
The activity of phosphatidylserine (PS)-exposing MVs (Zymuphen MP-activity assay). Plasmas from patients with pancreatic cancer have significantly more PS-exposing MVs than plasmas from healthy controls (n = 13, * indicates a significant difference between the two groups, unpaired t-test with Welch correction). Lines indicate mean PS equivalents (nM).

The activity of PS-exposing MVs was significantly higher in plasma from patients with pancreatic cancer than in healthy controls, with a mean (SD) of 57 (44) and 7 (2) nM PS equivalents, respectively (Fig 4, p = 0.001, n = 13). For the pancreatic cancer patients (n = 13), the association between the PS equivalents and the TG-parameters were: Peak (r = 0.71, p = 0.007), lag time (r = -0.60, p = 0.03), Velocity Index (r = 0.61, p = 0.03) and ETP (r = 0.41, p = 0.17) (S2 Fig).

## Discussion

In this study, we present the impact of the analytical variables CTI, anti-TFPI-abs and phospholipids (MP-reagent) on MV-associated thrombin generation in patients with pancreatic cancer and in healthy controls. PNP prepared without CTI in blood collection tubes with low contact activation, and inhibiting abs against TFPI added, offered the best assay conditions to measure MV-associated thrombin generation in the two groups. In our study, most of the MV-samples generated too low amounts of thrombin during native conditions (i.e. measurements without the addition of an MP-reagent or anti-TFPI abs). The thrombin generation curves did not reach the baseline, which made the LT the only objective parameter to evaluate. Furthermore, native MV-associated thrombin generation also gave numerous LTs > 20 min, where the duplicate CVs were relatively high. Others have reported anti-TFPI antibodies to increase TF-dependent thrombin generation of MVs generated *in vitro* [29, 32]. However, in our patient samples, the thrombin generation was still too low after the addition of anti-TFPI abs to PNP with CTI. Although recommended when measuring low levels of TF [28], the use of CTI in the PNP could mask the contribution from MV-associated molecules that activate FXII [35]. Previously, van Der Meijden *et al* have reported that *in vivo*-generated MVs from platelets and erythrocytes can initiate and propagate thrombin independently of TF in a FXII-dependent manner [36]. Recently, Nickel *et al* showed that extracellular vesicle (EV)-associated poly-phosphates (poly-P) in plasma from prostate cancer patients initiate coagulation in a FXII-dependent manner [37]. They also show that prostate-, pancreatic- and promyelocytic cell lines shed vesicles that are able to initiate coagulation in a similar manner [37].

The Monovette blood collection tubes we used for preparation of PNP have previously been reported to induce minimal contact activation compared to other commercially available tubes [38]. In accordance with this, we found that the native MV-associated thrombin generation in the healthy control group was low and quite similar in PNP with and without CTI. In contrast to MVs from healthy controls, MVs from pancreatic cancer patients seemed to generate more thrombin in the absence of CTI than in the presence of CTI, which could indicate a contribution from MVs that affect the intrinsic side of the coagulation cascade. Despite too low thrombin generation during these conditions, the overall shape of the thrombin generation curves seemed to discriminate the MV-associated thrombin generation between the two groups. However, adequate thrombin generation, i.e. all curves reached the baseline, was only achieved when CTI was omitted and anti-TFPI abs were added in the PNP. The presence of anti-TFPI abs increased the MV-associated thrombin generation in both groups, and we found significant differences between the pancreatic cancer patients and the healthy controls for all thrombin generation parameters. Notably, the difference in thrombin generation between the two groups were reduced or eliminated when synthetic phospholipids (MP-reagent) were added. With the Zymuphen MP-activity assay, we found that endogenous MV-bound PS levels were highly elevated in pancreatic cancer patients compared with healthy controls. Based on these two observations, we believe that the addition of an MP-reagent mask the dissimilar contribution of endogenous procoagulant MV-bound PS from the two groups. In addition, in the absence of CTI, the MP-reagent is able to amplify potential unspecific contact activation even when low contact-activating tubes are used [39].

The boosting effect of anti-TFPI abs was much more pronounced in the absence of CTI, which may indicate that the anti-TFPI abs allows amplification of MV-associated molecules that affects the intrinsic pathway. It is well known that TFPIα affects the extrinsic pathway by inhibiting the TF-FVIIa complex in a FXa-dependent manner [31], but TFPIα has also been reported to downregulate the initial phase of coagulation in a TF-independent manner through a protein S-dependent direct inhibition of FXa [40]. Also, TFPIα may inhibit prothrombinase through an interaction between the TFPIα C-terminus and the FV acidic region that is retained in early, FXa-activated FV [41].

With our optimized assay conditions (-CTI, -PS, +anti-TFPI abs), we showed a significantly different procoagulant potential of MVs from patients with pancreatic cancer compared with MVs from healthy individuals. In comparison, van Doormaal *et al* did not report significant differences in fibrin generation time between cancer patients and healthy individuals [17], and Thaler *et al* reported that MVs from patients with metastatic pancreatic cancer had a limited effect on time to fibrin clot formation despite a significantly higher MV-associated TF-activity as measured with a chromogenic, factor Xa-based assay [42]. However, these discrepancies may be explained by different experimental conditions like input volume and fibrin *vs* thrombin generation. Also, during our conditions it is possible to evaluate the whole thrombin generation curve, rather than just the initiation of coagulation. Endogenous PS, as well as other MV-associated molecules such as polyphosphates, may amplify the overall thrombin generation response. Therefore, it is important to also estimate other parameters than the LT/time to the initiation of fibrin clot. Compared with healthy controls, the level of MV-associated PS was highly elevated in our pancreatic cancer group. This is in contrast to the study by Thaler *et al* [42], but in accordance with other studies that have reported elevated levels of MV-bound PS in cancer patients, compared with healthy controls [16, 17, 43].

The contribution of TF to the MV-associated thrombin generation was analyzed in CTI-containing PNP, which should be the optimal condition for the detection of low TF. Except for in one patient with pancreatic cancer, we did not find MV-associated thrombin generation that was clearly dependent on TF across the four assay conditions. Of note, none of the patients with pancreatic cancer had clinical symptoms of disseminated intravascular coagulation (DIC) or ongoing thrombotic complications at the time point of blood collection. Other studies have reported increased MV-bound TF activity in cancer patients with thrombotic complications [16, 17, 44], rather than in the cancer group as such.

There are limitations to our study that should be mentioned. The plasma from the pancreatic cancer patients was stored 2–3 years longer than the plasma from healthy controls. Yuana *et al* recently reported that the concentration of PS-positive vesicles in plasma was not affected after one year of storage, whereas a 7-fold increase in the number of PS-exposing EVs was found after a single freeze-thaw cycle [45]. Our samples were immediately frozen after blood collection and thawed just before MV-preparation, and possible increase in PS-exposing MVs would therefore apply for both the pancreatic cancer group and the healthy control group. The use of isolated MVs in our study eliminates possible plasma interferences such as deficiency of coagulation factors and treatment with anticoagulant drugs. However, it will also eliminate regulatory activators/inhibitors present in each individual patient’s plasma, and therefore measure the isolated procoagulant potential of the MVs. In this study, our aim has been to use a small number of samples from pancreatic cancer patients and healthy controls to present the impact of analytical variables on MV-associated thrombin generation in clinical samples. Obviously, larger studies are needed in order to evaluate this modified assay in the measurements of the procoagulant potential of circulating MVs in different diseases and individual patients.

The most favorable analytical condition to measure MV-associated thrombin generation in patients with pancreatic cancer and healthy controls was to add the isolated MVs into low contact-activated PNP without CTI and without external phospholipids, but in the presence of anti-TFPI antibodies. These assay conditions gave good repeatability, and the contribution from MVs affecting both the extrinsic and intrinsic pathway can be measured. Although demonstrated in samples from patients with meningococcal sepsis, the ability to detect MV- associated TF-activity (by adding anti-TF abs) in diseases with low TF, remains to be elucidated. This modified thrombin generation assay could be a valuable contribution to the existing methods for measuring circulating MVs in conditions with increased risk of thrombotic complications.

## Supporting information

S1 Fig The effect of inhibiting antibodies (abs) against tissue factor (TF) on microvesicle (MV)-associated thrombin generation (lag time) in samples from patients with meningococcal sepsis. MVs were preincubated with or without anti-TF abs, and analyzed in pooled normal plasma (PNP) without corn trypsin inhibitor (CTI), but with anti-TFPI abs added.
(TIF)

S2 Fig The associations between thrombin generation (TG) parameters and PS equivalents. The associations between the TG parameters Peak, lag time, velocity index and ETP for the pancreatic cancer patients samples are presented (Pearson’s test, n = 13).
(TIF)

S1 TableCV of duplicate measurements–raw data and formulas.CV calculations are based on duplicate measurements (x_1_ and x_2_) in samples from both the pancreatic cancer patients (P1-P13) and the healthy controls (HC1-HC13). In the experiments performed in PNP with CTI, the results from the samples incubated with anti-TF abs were also included (n = 52). The formulas used are:
$$Mean=\frac{\sum \left(x_{1}+x_{2}\right)}{2n}$$
$$SD=\surd \frac{\sum {\left(x_{1}-x_{2}\right)}^{2}}{2n}$$
$CV\;(\%)=100\;x\;\frac{SD}{Mean}$(PDF)

## References

[1] Pabinger I, Thaler J, Ay C. Biomarkers for prediction of venous thromboembolism in cancer. Blood. 2013;122(12):2011–8. Epub 2013/08/03. doi: 10.1182/blood-2013-04-460147 23908470 10.1182/blood-2013-04-460147

[2] Campello E, Spiezia L, Radu CM, Simioni P. Microparticles as biomarkers of venous thromboembolic events. Biomark Med. 2016;10(7):743–55. Epub 2016/06/25. doi: 10.2217/bmm-2015-0063 27338783 10.2217/bmm-2015-0063

[3] Gyorgy B, Szabo TG, Pasztoi M, Pal Z, Misjak P, Aradi B, et al Membrane vesicles, current state-of-the-art: emerging role of extracellular vesicles. Cell Mol Life Sci. 2011;68(16):2667–88. Epub 2011/05/12. doi: 10.1007/s00018-011-0689-3 21560073 10.1007/s00018-011-0689-3 PMC3142546

[4] Freyssinet JM, Toti F. Formation of procoagulant microparticles and properties. Thromb Res. 2010;125 Suppl 1:S46–8. Epub 2010/02/16. doi: 10.1016/j.thromres.2010.01.036 20153515 10.1016/j.thromres.2010.01.036

[5] Morel O, Jesel L, Freyssinet JM, Toti F. Cellular mechanisms underlying the formation of circulating microparticles. Arterioscler Thromb Vasc Biol. 2011;31(1):15–26. Epub 2010/12/17. doi: 10.1161/ATVBAHA.109.200956 21160064 10.1161/ATVBAHA.109.200956

[6] Rapaport SI, Rao LV. Initiation and regulation of tissue factor-dependent blood coagulation. Arterioscler Thromb. 1992;12(10):1111–21. Epub 1992/10/01. 1390583 10.1161/01.atv.12.10.1111

[7] Han X, Guo B, Li Y, Zhu B. Tissue factor in tumor microenvironment: a systematic review. J Hematol Oncol. 2014;7:54 Epub 2014/08/03. doi: 10.1186/s13045-014-0054-8 25084809 10.1186/s13045-014-0054-8 PMC4237870

[8] Khorana AA, Ahrendt SA, Ryan CK, Francis CW, Hruban RH, Hu YC, et al Tissue factor expression, angiogenesis, and thrombosis in pancreatic cancer. Clin Cancer Res. 2007;13(10):2870–5. Epub 2007/05/17. doi: 10.1158/1078-0432.CCR-06-2351 17504985 10.1158/1078-0432.CCR-06-2351

[9] Seto S, Onodera H, Kaido T, Yoshikawa A, Ishigami S, Arii S, et al Tissue factor expression in human colorectal carcinoma: correlation with hepatic metastasis and impact on prognosis. Cancer. 2000;88(2):295–301. Epub 2000/01/21. 10640960 10.1002/(sici)1097-0142(20000115)88:2<295::aid-cncr8>3.0.co;2-u

[10] Ueno T, Toi M, Koike M, Nakamura S, Tominaga T. Tissue factor expression in breast cancer tissues: its correlation with prognosis and plasma concentration. Br J Cancer. 2000;83(2):164–70. Epub 2000/07/20. doi: 10.1054/bjoc.2000.1272 10901365 10.1054/bjoc.2000.1272 PMC2363475

[11] Rautou PE, Mackman N. Microvesicles as risk markers for venous thrombosis. Expert Rev Hematol. 2013;6(1):91–101. Epub 2013/02/05. doi: 10.1586/ehm.12.74 23373784 10.1586/ehm.12.74

[12] Khorana AA, Fine RL. Pancreatic cancer and thromboembolic disease. Lancet Oncol. 2004;5(11):655–63. Epub 2004/11/04. doi: 10.1016/S1470-2045(04)01606-7 15522652 10.1016/S1470-2045(04)01606-7

[13] Thaler J, Ay C, Mackman N, Bertina RM, Kaider A, Marosi C, et al Microparticle-associated tissue factor activity, venous thromboembolism and mortality in pancreatic, gastric, colorectal and brain cancer patients. J Thromb Haemost. 2012;10(7):1363–70. Epub 2012/04/24. doi: 10.1111/j.1538-7836.2012.04754.x 22520016 10.1111/j.1538-7836.2012.04754.x

[14] Bharthuar A, Khorana AA, Hutson A, Wang JG, Key NS, Mackman N, et al Circulating microparticle tissue factor, thromboembolism and survival in pancreaticobiliary cancers. Thromb Res. 2013;132(2):180–4. Epub 2013/07/17. doi: 10.1016/j.thromres.2013.06.026 23856554 10.1016/j.thromres.2013.06.026

[15] Thaler J, Ay C, Mackman N, Metz-Schimmerl S, Stift J, Kaider A, et al Microparticle-associated tissue factor activity in patients with pancreatic cancer: correlation with clinicopathological features. Eur J Clin Invest. 2013;43(3):277–85. Epub 2013/02/13. doi: 10.1111/eci.12042 23398637 10.1111/eci.12042

[16] Tesselaar ME, Romijn FP, Van Der Linden IK, Prins FA, Bertina RM, Osanto S. Microparticle-associated tissue factor activity: a link between cancer and thrombosis? J Thromb Haemost. 2007;5(3):520–7. Epub 2006/12/15. doi: 10.1111/j.1538-7836.2007.02369.x 17166244 10.1111/j.1538-7836.2007.02369.x

[17] van Doormaal F, Kleinjan A, Berckmans RJ, Mackman N, Manly D, Kamphuisen PW, et al Coagulation activation and microparticle-associated coagulant activity in cancer patients. An exploratory prospective study. Thromb Haemost. 2012;108(1):160–5. Epub 2012/04/27. doi: 10.1160/TH12-02-0099 22535219 10.1160/TH12-02-0099

[18] Khorana AA, Francis CW, Menzies KE, Wang JG, Hyrien O, Hathcock J, et al Plasma tissue factor may be predictive of venous thromboembolism in pancreatic cancer. J Thromb Haemost. 2008;6(11):1983–5. Epub 2008/09/18. doi: 10.1111/j.1538-7836.2008.03156.x 18795992 10.1111/j.1538-7836.2008.03156.x PMC2848502

[19] Pabinger I, Posch F. Flamethrowers: blood cells and cancer thrombosis risk. Hematology Am Soc Hematol Educ Program. 2014;2014(1):410–7. Epub 2015/02/20. doi: 10.1182/asheducation-2014.1.410 25696887 10.1182/asheducation-2014.1.410

[20] Owens AP 3rd, Mackman N. Microparticles in hemostasis and thrombosis. Circ Res. 2011;108(10):1284–97. Epub 2011/05/14. doi: 10.1161/CIRCRESAHA.110.233056 21566224 10.1161/CIRCRESAHA.110.233056 PMC3144708

[21] Mooberry MJ, Key NS. Microparticle analysis in disorders of hemostasis and thrombosis. Cytometry Part A: the journal of the International Society for Analytical Cytology. 2015 Epub 2015/02/24. doi: 10.1002/cyto.a.22647 25704723 10.1002/cyto.a.22647 PMC4545474

[22] Key NS, Mackman N. Tissue factor and its measurement in whole blood, plasma, and microparticles. Semin Thromb Hemost. 2010;36(8):865–75. Epub 2010/11/05. doi: 10.1055/s-0030-1267040 21049387 10.1055/s-0030-1267040

[23] Hemker HC, Giesen P, Al Dieri R, Regnault V, de Smedt E, Wagenvoord R, et al Calibrated automated thrombin generation measurement in clotting plasma. Pathophysiol Haemost Thromb. 2003;33(1):4–15. Epub 2003/07/11. doi: 71636 12853707 10.1159/000071636

[24] Ay C, Dunkler D, Simanek R, Thaler J, Koder S, Marosi C, et al Prediction of venous thromboembolism in patients with cancer by measuring thrombin generation: results from the Vienna Cancer and Thrombosis Study. J Clin Oncol. 2011;29(15):2099–103. Epub 2011/04/06. doi: 10.1200/JCO.2010.32.8294 21464402 10.1200/JCO.2010.32.8294

[25] Bidot L, Jy W, Bidot C Jr., Jimenez JJ, Fontana V, Horstman LL, et al Microparticle-mediated thrombin generation assay: increased activity in patients with recurrent thrombosis. J Thromb Haemost. 2008;6(6):913–9. Epub 2008/03/28. doi: 10.1111/j.1538-7836.2008.02963.x 18363818 10.1111/j.1538-7836.2008.02963.x

[26] Aleman MM, Gardiner C, Harrison P, Wolberg AS. Differential contributions of monocyte- and platelet-derived microparticles towards thrombin generation and fibrin formation and stability. J Thromb Haemost. 2011;9(11):2251–61. Epub 2011/09/03. doi: 10.1111/j.1538-7836.2011.04488.x 21883880 10.1111/j.1538-7836.2011.04488.x PMC3206146

[27] Gheldof D, Hardij J, Cecchet F, Chatelain B, Dogne JM, Mullier F. Thrombin generation assay and transmission electron microscopy: a useful combination to study tissue factor-bearing microvesicles. Journal of extracellular vesicles. 2013;2 doi: 10.3402/jev.v2i0.19728 24009889 10.3402/jev.v2i0.19728 PMC3760633

[28] Luddington R, Baglin T. Clinical measurement of thrombin generation by calibrated automated thrombography requires contact factor inhibition. J Thromb Haemost. 2004;2(11):1954–9. Epub 2004/11/20. doi: 10.1111/j.1538-7836.2004.00964.x 15550027 10.1111/j.1538-7836.2004.00964.x

[29] Gheldof D, Mullier F, Chatelain B, Dogne JM, Chatelain C. Inhibition of tissue factor pathway inhibitor increases the sensitivity of thrombin generation assay to procoagulant microvesicles. Blood Coagul Fibrinolysis. 2013;24(5):567–72. doi: 10.1097/MBC.0b013e328360a56e 23807485 10.1097/MBC.0b013e328360a56e

[30] Brodin E, Appelbom H, Osterud B, Hilden I, Petersen LC, Hansen JB. Regulation of thrombin generation by TFPI in plasma without and with heparin. Transl Res. 2009;153(3):124–31. Epub 2009/02/17. doi: 10.1016/j.trsl.2008.12.004 19218095 10.1016/j.trsl.2008.12.004

[31] Wood JP, Ellery PE, Maroney SA, Mast AE. Biology of tissue factor pathway inhibitor. Blood. 2014;123(19):2934–43. Epub 2014/03/13. doi: 10.1182/blood-2013-11-512764 24620349 10.1182/blood-2013-11-512764 PMC4014837

[32] Keuren JF, Magdeleyns EJ, Govers-Riemslag JW, Lindhout T, Curvers J. Effects of storage-induced platelet microparticles on the initiation and propagation phase of blood coagulation. Br J Haematol. 2006;134(3):307–13. Epub 2006/07/20. doi: 10.1111/j.1365-2141.2006.06167.x 16848773 10.1111/j.1365-2141.2006.06167.x

[33] Brummel-Ziedins KE, Vossen CY, Butenas S, Mann KG, Rosendaal FR. Thrombin generation profiles in deep venous thrombosis. J Thromb Haemost. 2005;3(11):2497–505. Epub 2005/10/26. doi: 10.1111/j.1538-7836.2005.01584.x 16241948 10.1111/j.1538-7836.2005.01584.x PMC1410192

[34] Hellum M, Ovstebo R, Brusletto BS, Berg JP, Brandtzaeg P, Henriksson CE. Microparticle-associated tissue factor activity correlates with plasma levels of bacterial lipopolysaccharides in meningococcal septic shock. Thromb Res. 2014;133(3):507–14. Epub 2014/01/16. doi: 10.1016/j.thromres.2013.12.031 24423888 10.1016/j.thromres.2013.12.031

[35] Kluft C, Meijer P. External quality assessment for thrombin generation tests: an exploration. Semin Thromb Hemost. 2010;36(7):791–6. Epub 2010/10/28. doi: 10.1055/s-0030-1265296 20979000 10.1055/s-0030-1265296

[36] Van Der Meijden PE, Van Schilfgaarde M, Van Oerle R, Renne T, ten Cate H, Spronk HM. Platelet- and erythrocyte-derived microparticles trigger thrombin generation via factor XIIa. J Thromb Haemost. 2012;10(7):1355–62. Epub 2012/04/28. doi: 10.1111/j.1538-7836.2012.04758.x 22537188 10.1111/j.1538-7836.2012.04758.x

[37] Nickel KF, Ronquist G, Langer F, Labberton L, Fuchs TA, Bokemeyer C, et al The polyphosphate-factor XII pathway drives coagulation in prostate cancer-associated thrombosis. Blood. 2015;126(11):1379–89. Epub 2015/07/15. doi: 10.1182/blood-2015-01-622811 26153520 10.1182/blood-2015-01-622811 PMC4566812

[38] Ramstrom S. Clotting time analysis of citrated blood samples is strongly affected by the tube used for blood sampling. Blood Coagul Fibrinolysis. 2005;16(6):447–52. Epub 2005/08/12. 16093737 10.1097/01.mbc.0000178827.52242.89

[39] Boknas N, Faxalv L, Lindahl TL, Ramstrom S. Contact activation: important to consider when measuring the contribution of tissue factor-bearing microparticles to thrombin generation using phospholipid-containing reagents. J Thromb Haemost. 2014;12(4):515–8. Epub 2014/01/11. doi: 10.1111/jth.12503 24405583 10.1111/jth.12503

[40] Thomassen MC, Heinzmann AC, Herfs L, Hartmann R, Dockal M, Scheiflinger F, et al Tissue factor-independent inhibition of thrombin generation by tissue factor pathway inhibitor-alpha. J Thromb Haemost. 2015;13(1):92–100. Epub 2014/10/29. doi: 10.1111/jth.12766 25348176 10.1111/jth.12766

[41] Wood JP, Bunce MW, Maroney SA, Tracy PB, Camire RM, Mast AE. Tissue factor pathway inhibitor-alpha inhibits prothrombinase during the initiation of blood coagulation. Proc Natl Acad Sci U S A. 2013;110(44):17838–43. Epub 2013/10/16. doi: 10.1073/pnas.1310444110 24127605 10.1073/pnas.1310444110 PMC3816478

[42] Thaler J, Koder S, Kornek G, Pabinger I, Ay C. Microparticle-associated tissue factor activity in patients with metastatic pancreatic cancer and its effect on fibrin clot formation. Transl Res. 2014;163(2):145–50. Epub 2013/08/27. doi: 10.1016/j.trsl.2013.06.009 23973167 10.1016/j.trsl.2013.06.009

[43] Thaler J, Ay C, Weinstabl H, Dunkler D, Simanek R, Vormittag R, et al Circulating procoagulant microparticles in cancer patients. Ann Hematol. 2011;90(4):447–53. Epub 2010/10/29. doi: 10.1007/s00277-010-1111-1 20981426 10.1007/s00277-010-1111-1

[44] Tesselaar ME, Romijn FP, van der Linden IK, Bertina RM, Osanto S. Microparticle-associated tissue factor activity in cancer patients with and without thrombosis. J Thromb Haemost. 2009;7(8):1421–3. Epub 2009/06/09. doi: 10.1111/j.1538-7836.2009.03504.x 19500241 10.1111/j.1538-7836.2009.03504.x

[45] Yuana Y, Boing AN, Grootemaat AE, van der Pol E, Hau CM, Cizmar P, et al Handling and storage of human body fluids for analysis of extracellular vesicles. Journal of extracellular vesicles. 2015;4:29260 Epub 2015/11/14. doi: 10.3402/jev.v4.29260 26563735 10.3402/jev.v4.29260 PMC4643195

