# Supplementary figures and images for: The effect of corn trypsin inhibitor, anti-tissue factor pathway inhibitor antibodies and phospholipids on microvesicle-associated thrombin generation in patients with pancreatic cancer and healthy controls
Source: PLoS One. 2017 Sep 14;12(9):e0184579. doi: 10.1371/journal.pone.0184579 (PMC5598995; doi:10.1371/journal.pone.0184579)

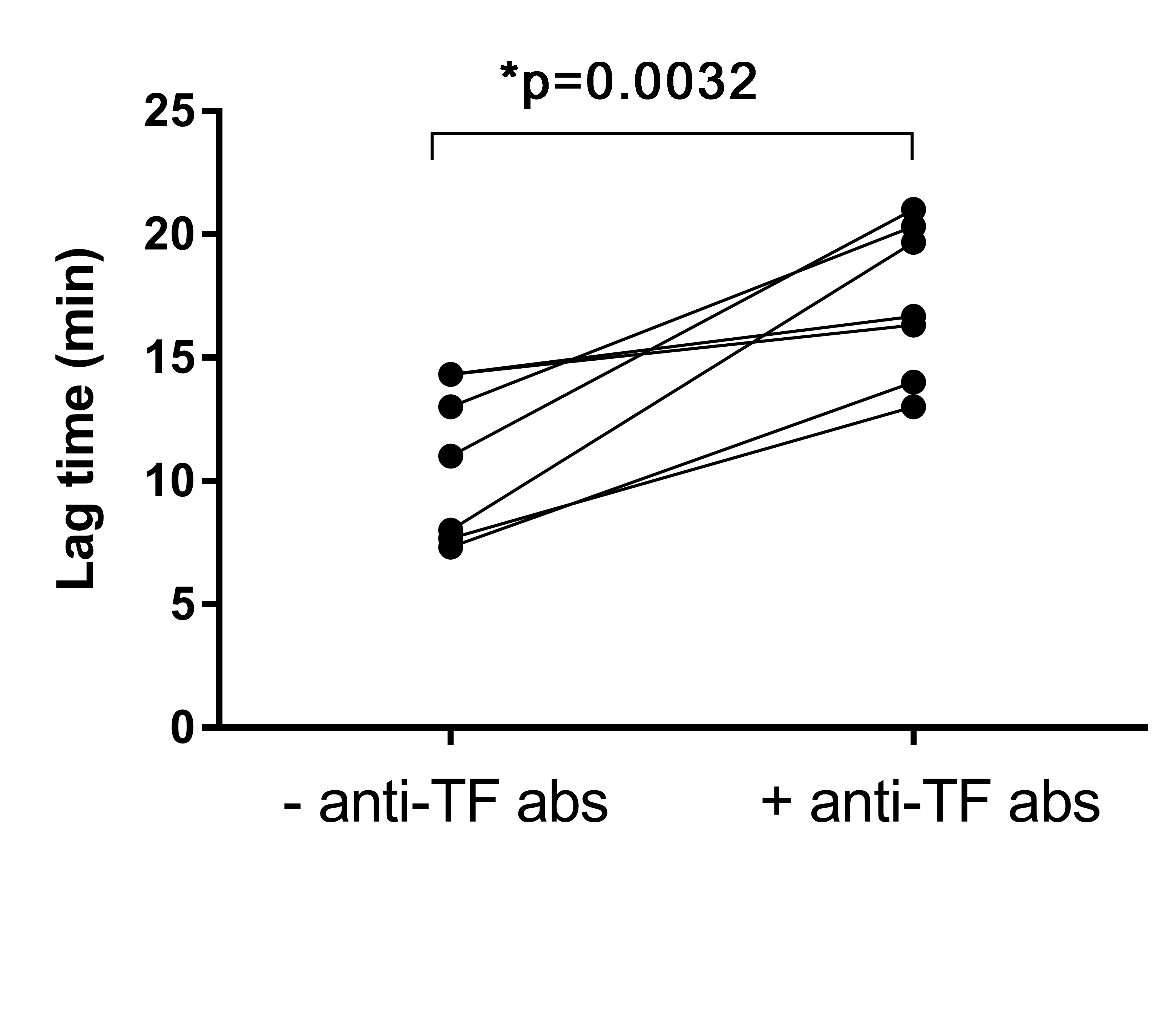

Supplement: S1 Fig — MVs were preincubated with or without anti-TF abs, and analyzed in pooled normal plasma (PNP) without corn trypsin inhibitor (CTI), but with anti-TFPI abs added. (TIF) [file pone.0184579.s001.tif]

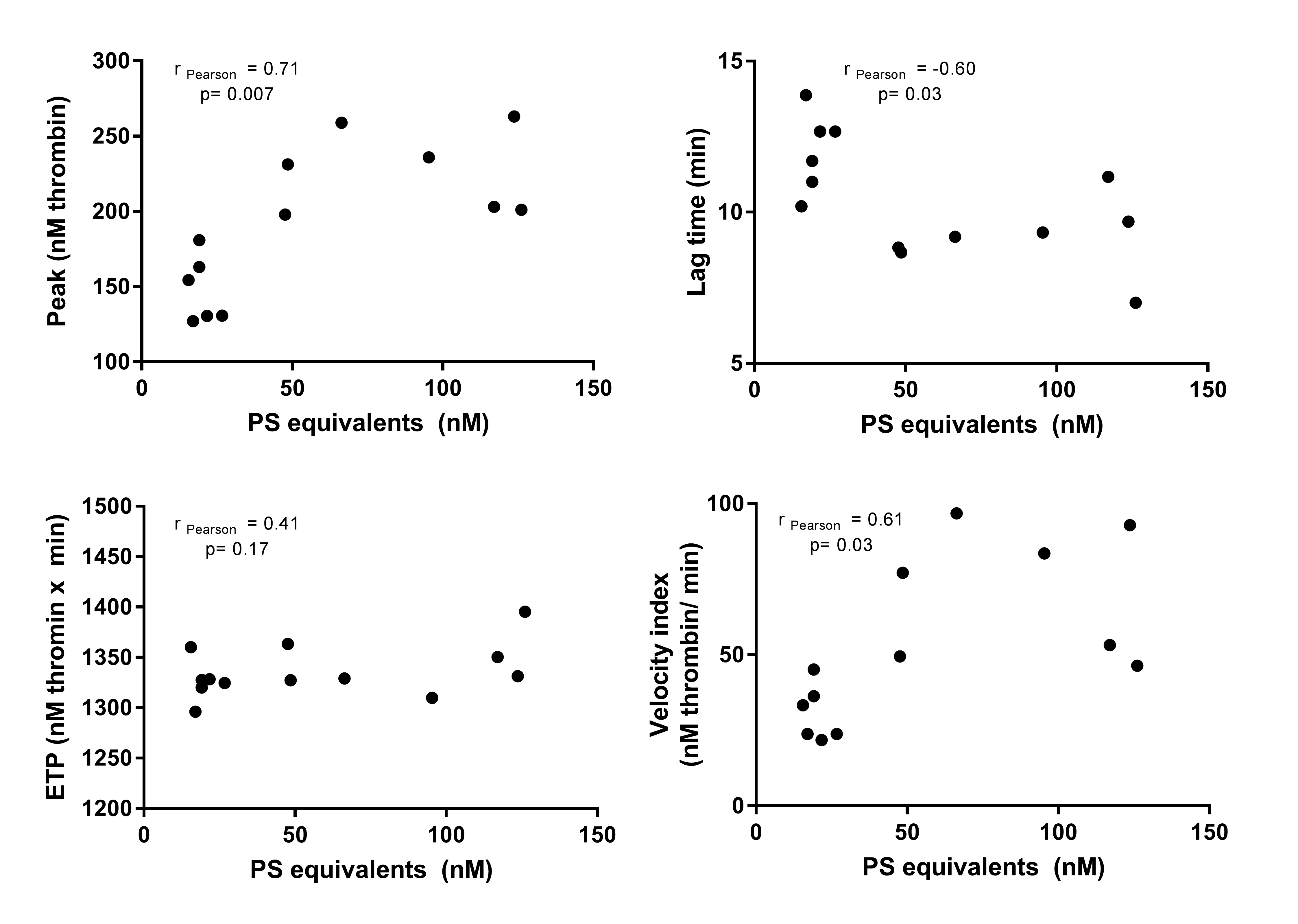

Supplement: S2 Fig — The associations between the TG parameters Peak, lag time, velocity index and ETP for the pancreatic cancer patients samples are presented (Pearson’s test, n = 13). (TIF) [file pone.0184579.s002.tif]
