# Supplementary material for: The effect of corn trypsin inhibitor, anti-tissue factor pathway inhibitor antibodies and phospholipids on microvesicle-associated thrombin generation in patients with pancreatic cancer and healthy controls
Source: PLoS One. 2017 Sep 14;12(9):e0184579. doi: 10.1371/journal.pone.0184579 (PMC5598995; doi:10.1371/journal.pone.0184579)
Supplement: S1 Table — CV calculations are based on duplicate measurements (x1 and x2) in samples from both the pancreatic cancer patients (P1-P13) and the healthy controls (HC1-HC13). In the experiments performed in PNP with CTI, the results from the samples incubated with anti-TF abs were also included (n = 52). The formulas used are: Mean=∑(x1+x2)2n SD=√∑(x1−x2)22n CV(%)=100xSDMean (PDF) [file pone.0184579.s003.pdf]

	+CTI, - MP reagent, - anti-TF abs				+CTI, - MP reagent, + anti-TF abs			
	-anti-TFPI abs		+anti-TFPI abs		-anti-TFPI abs		+anti-TFPI abs	
	Lag time (min)				Lag time (min)			
	X_1	X_2	X_1	X_2	X_1	X_2	X_1	X_2
P1	20,3	18,0	13,6	15,3	19,6	19,3	17,3	17,0
P2	18,6	19,6	14,6	15,0	17,0	14,0	16,0	13,3
P3	18,3	17,0	13,0	13,0	19,7	19,0	14,0	14,0
P4	18,7	18,3	14,0	14,0	19,0	18,7	14,0	14,3
P5	19,7	21,0	15,3	15,0	23,3	20,0	16,7	17,0
P6	20,3	23,7	15,0	15,0	21,3	17,3	18,7	17,7
P7	15,7	15,0	11,7	12,0	14,0	14,7	13,0	13,0
P8	15,7	14,3	12,7	13,3	16,0	18,3	14,0	14,0
P9	16,3	14,3	13,0	12,7	15,7	17,0	13,0	13,3
P10	11,7	11,0	11,3	10,7	13,7	12,3	11,3	11,7
P11	13,7	14,3	10,7	12,0	15,3	13,3	10,3	12,0
P12	10,7	11,0	8,7	9,0	16,7	17,3	13,0	13,0
P13	19,7	19,0	15,3	15,0	16,7	16,3	16,0	16,0
HC1	24,3	19,6	20,0	20,0	33,0	31,0	20,7	21,0
HC2	13,6	13,6	13,6	12,3	15,3	14,6	12,0	14,0
HC3	14,3	14,7	14,7	14,3	12,7	18,0	13,3	14,7
HC4	27,3	41,7	18,7	24,3	31,3	33,3	18,7	21,0
HC5	44,7	26,3	19,3	24,3	33,0	37,7	18,3	25,3
HC6	32,0	34,7	20,3	19,0	43,0	38,0	25,3	25,3
HC7	25,7	34,7	20,7	21,3	34,7	33,7	22,7	23,3
HC8	18,0	26,7	17,3	20,3	37,0	31,7	23,0	17,7
HC9	13,3	13,3	12,0	12,0	12,3	15,0	13,7	14,0
HC10	16,0	20,0	17,0	15,3	16,7	16,0	14,0	15,7
HC11	19,0	19,0	16,7	17,0	18,7	15,3	14,7	17,3
HC12	33,3	24,3	22,3	23,0	33,0	34,7	21,3	24,3
HC13	32,3	25,0	21,0	21,7	33,3	31,3	22,7	21,7

	-CTI, - MP reagent									
	-anti-TFPI abs		+anti-TFPI abs		+anti-TFPI abs		+anti-TFPI abs		+anti-TFPI abs	
	Lag time (min)				ETP (nM thrombin x min)		Peak (nM thrombin)		Velocity index (nM thrombin/min)	
	X_1	X_2	X_1	X_2	X_1	X_2	X_1	X_2	X_1	X_2
P1	18,4	17,4	10,4	10,0	1377	1343	149	160	29,6	36,9
P2	16,4	26,1	11,7	11,7	1301	1354	179	183	44,5	45,7
P3	15,7	20,1	9,0	9,4	1324	1334	261	257	97,6	96,1
P4	22,7	13,7	9,7	9,7	1335	1328	265	262	98,9	86,9
P5	18,7	17,7	13,7	14,0	1291	1301	128	126	24,0	23,6
P6	20,7	24,0	9,7	15,7	1343	1307	149	112	29,8	17,7
P7	15,0	14,3	9,0	8,7	1347	1380	199	197	49,7	49,2
P8	16,7	18,3	11,0	11,0	1319	1322	165	161	38,1	34,6
P9	16,3	21,3	9,3	9,3	1297	1322	235	237	78,4	88,8
P10	12,0	10,7	8,3	9,0	1345	1310	234	228	78,0	76,2
P11	16,7	21,3	10,3	12,0	1340	1361	225	181	67,6	38,7
P12	10,3	9,7	7,3	6,7	1411	1380	199	203	45,9	46,9
P13	20,0	16,7	12,7	12,7	1321	1336	132	129	22,0	21,6
HC1	32,4	39,4	13,0	13,7	1173	1148	92	94	15,3	15,7
HC2	16,1	19,7	14,0	14,0	1184	1232	85	86	12,0	11,2
HC3	22,1	22,4	10,7	13,4	1320	1317	119	122	20,9	22,7
HC4	18,7	51,8	13,0	14,0	1092	1074	84	84	15,8	15,7
HC5	58,2	23,1	14,7	16,7	1028	1030	70	69	12,3	11,4
HC6	36,7	23,3	11,3	11,3	1168	1172	82	83	11,7	12,6
HC7	-	-	13,7	13,0	1063	1109	72	74	10,8	10,5
HC8	27,3	23,0	13,3	13,7	1156	1155	83	82	13,0	12,9
HC9	13,0	14,7	9,7	11,7	1082	1054	80	75	12,6	12,5
HC10	16,0	16,0	13,0	13,3	1140	1145	107	106	18,8	20,0
HC11	19,4	36,1	11,4	12,4	1284	1265	147	148	31,5	31,6
HC12	25,7	29,7	16,3	17,7	1083	1071	60	62	6,9	7,5
HC13	48,0	42,3	18,0	14,7	1127	1163	75	76	9,8	9,5
